# Characteristics and influencing factors of caregivers’ healthcare preferences for young children under COVID-19 lockdown: a cross-sectional study in Shanghai, China

**DOI:** 10.1186/s12875-024-02484-4

**Published:** 2024-07-20

**Authors:** Wenya Yu, Jiahe Tian, Panpan Li, Zhichao Guo, Dan ZCM, Meina Li, Yang Ge, Xiang Liu

**Affiliations:** 1 School of Public Health, Shanghai Jiao Tong University School of Medicine, Shanghai, 200025 China; 2 Department of Prevention and Health Care, Dapuqiao Community Health Service Center of Huangpu District, Shanghai, 200001 China; 3 Department of Prevention and Health Care, Yuepu Town Community Health Service Center of Baoshan District, Shanghai, 200941 China; 4 Department of Military Medical Service, Faculty of Military Health Service, Naval Medical University, Shanghai, 200433 China; 5 Affiliated Xihu Hospital, Hangzhou Medical College, Hangzhou, 310000 China; 6 Department of Respiratory Disease, The 903rd Hospital of PLA, Hangzhou, 310000 China

**Keywords:** Children, COVID-19, Healthcare, Pediatric, Healthcare preference

## Abstract

**Background:**

Missed or delayed child healthcare caused by the COVID-19 lockdown has threatened young children’s health and has had an unpredictable influence on caregivers’ child healthcare preferences. This study investigated caregivers’ child healthcare preferences and the factors that influence them among families with young children (0–3 years) during the lockdown in Shanghai.

**Methods:**

Participants in this cross-sectional study were enrolled through random encounter sampling. Questionnaires were distributed online from June 1 to November 10, 2022, in Shanghai. A total of 477 valid questionnaires were received. The demographics of caregivers and their families, children’s characteristics, COVID-19-related information, and caregivers’ healthcare preferences were analyzed. The statistical analyses included frequency and percentage, chi-square tests, and multinomial logistic regression.

**Results:**

Caregivers preferred child healthcare professionals in the community health service system (CHS; 47.6%) followed by hospital pediatricians (40.0%) during lockdown. Caregivers with the following characteristics preferred CHS: those with an annual household income of CNY 200,000–300,000, those whose youngest children were aged 8–12 months, and those who experienced early childhood physical development issues. Caregivers preferred hospitals if they had experienced healthcare-seeking-related difficulties in accessing professional guidance from hospital pediatricians.

**Conclusions:**

During pandemic lockdowns, policymakers should allocate more resources to CHS to meet caregivers’ childcare demands. Moreover, special attention should be given to the healthcare needs for CHS among families with specific demographics.

**Trial registration:**

Approval was obtained from the Ethics Committee of Shanghai Jiao Tong University School of Medicine School of Public Health (SJUPN-202,109; June 1, 2022).

**Supplementary Information:**

The online version contains supplementary material available at 10.1186/s12875-024-02484-4.

## Background

Apart from disease diagnosis and treatment, there are specific healthcare requirements for early childhood development, especially for children aged 3 years and under [1]. In China, children under 3 years of age must complete regular physical growth and developmental check-ups and planned vaccinations, and their caregivers’ have high expectations of professional child-rearing consultation and guidance [2, 3]. Moreover, the completion of these check-ups is one of the entry requirements for kindergarten. Child healthcare services in China are delivered through the three-level public healthcare system. This system comprises primary healthcare institutions, secondary hospitals, tertiary hospitals, and private healthcare institutions [4]. The primary healthcare institutions comprise the community health service (CHS) system in urban areas and healthcare clinics in rural areas. In Shanghai, for example, the public healthcare system, which includes the CHS, secondary, and tertiary hospitals, offers the majority of regular services for young children under 3 years of age. These services encompass physical development check-ups, planned immunization, and professional guidance on child nutrition and parenting. By contrast, private childcare institutions generally meet higher-level healthcare demands. Caregivers typically have the freedom to choose any healthcare institution they prefer for child healthcare. In 2019, child healthcare departments in CHS, pediatric departments in secondary and tertiary hospitals, and private childcare institutions in Shanghai collectively conducted 12.905 million consultations, with the respective proportions being 54.0%, 45.9%, and 0.1% [5].

However, the COVID-19 pandemic led directly to unprecedented challenges for health and healthcare fields owing to the lockdown measures, which were particularly severe on pediatric healthcare [6, 7]. During the lockdown, evidence from various countries demonstrated that the aforementioned childcare services were unavailable [8, 9], and the primary healthcare system in many regions, including China, was virtually stagnant, with reduced health check-ups, follow-ups, and guidance [10, 11]. As one of China’s megacities, Shanghai also underwent lockdown measures. The city experienced a long-term, large-scale outbreak, with lockdown measures in place up to early 2022. Specifically, the lockdown lasted from March 28 to June 1, 2022, with approximately 57,595 cases of infection and 581 deaths recorded [12].

As a result, most healthcare services including childcare, were significantly reduced, or suspended due to inaccessible or inadequate healthcare resources. For example, 75.2% of counties and cities assigned COVID-19 related responsibilities to personnel who should have been providing primary healthcare at the Centers for Disease Control and primary healthcare institutions [13]; many clinical departments in hospitals were converted to COVID-19 wards [14, 15]; and the number of healthcare consultations decreased by 71% and much more severely in the economically-developed regions (e.g., Shanghai) [13]. In an attempt to guarantee child health during lockdown, social support was provided for reopening child healthcare services during lockdown (e.g., releasing healthcare resources occupied by lockdown, restoring pediatric healthcare services in hospitals, and reopening pediatric follow-up visits) for young children under 3 years of age at CHS and hospitals [16]. However, missed or delayed routine check-ups, immunizations, early childhood development consultations and interventions, and common disease diagnoses and treatments were still great threats due to the personal factors of caregivers. For example, caregivers reduced child healthcare visits, especially for children aged 0–3 years, as they feared COVID-19 exposure [17–19]. Therefore, an unreasonable healthcare resource allocation and provision resulted due to a failure to match healthcare provision to healthcare preferences. This not only wasted limited healthcare resources during the lockdown, but also negatively influenced children’s health outcomes [20].

Based on the social ecological model (SEM) [21, 22], which is a useful framework for predicting disease management behaviors and assumes health-seeking behaviors are determined by multiple factors, we hypothesized that under COVID-19 lockdown, caregivers’ healthcare preferences for young children differed from their preferences during normal times and were influenced by social support, personal factors, and the accessibility of healthcare resources. Furthermore, because existing studies mainly focused on the reduction in the utilization of child healthcare during the lockdown [8, 23, 24], less was known about caregivers’ healthcare preferences for their young children.

Given the unclear influence of COVID-19 lockdown on caregivers’ healthcare preferences, which determine optimal medical resource allocation and children’s health outcomes, this study aimed to investigate caregivers’ healthcare preferences for young children during the COVID-19 lockdown in Shanghai and explore related influencing factors. The findings can provide concrete strategies for medical resource allocation and service provision in China and other developing countries with similar healthcare systems to better respond to future large-scale pandemics. Additionally, considering the global nature of the pandemic [25] and the importance of medical resource allocation for emergency preparedness, this study could offer valuable analytical insights for other countries and regions to better allocate limited medical resources, which is crucial for early preparation for future pandemics.

## Method

### Study design

This study was conducted via an online survey, which explored caregivers’ healthcare preferences for children aged 0–3 years and the factors influencing those preferences under the COVID-19 lockdown in Shanghai, China. This study had a minimum calculated sample size of 369, a confidence level of 95%, a permissible error of 0.1, and a COVID-19 prevalence rate of 2.0% in young children [26]. As the questionnaire used in this study was self-designed and unpublished, it was necessary to evaluate its reliability and validity to ensure the accuracy of the findings. We assessed these metrics using Cronbach’s alpha value and the Kaiser–Meyer–Olkin (KMO) measure [27, 28]. Both values of 0.7 or higher are considered acceptable [29, 30]. The questionnaire’s Cronbach’s alpha value was 0.782, and the KMO was 0.780, indicating good reliability and validity. All variables, except socio-demographic characteristics, were included in these assessments.

The 24-item questionnaire contained four categories (Supplementary File. S1). The first category included caregiver and family demographics, including their relationship with their child, sex, age, education level, occupation, marital status, household income, and residential location. The second category included information on each family’s youngest child, such as number of siblings, age, preterm birth, low birth weight, neonatal intensive care unit admission, and feeding pattern. The third category included COVID-19-related information, such as the number of family members who co-resided during the lockdown, knowing an infected person, working from home, parenting issues, external difficulties, parenting anxiety, healthcare demand unavailability, and lack of physical growth and developmental check-ups and vaccinations. The fourth category included caregivers’ child healthcare preference information. In this category, we employed visually striking methods such as bolding text and changing font colors when requesting caregivers to provide information on their child healthcare preferences during lockdown.

### Data collection

Due to the ongoing pandemic control measures in China at the time of this study, conducting surveys based on community or administrative regions was challenging. To minimize the risk of infection from face-to-face contact, this study utilized convenience sampling [31]. Surveys were distributed online via social media platforms (e.g., WeChat), allowing open participation and invitations to others. However, only responses meeting the inclusion criteria were retained for the study. In the phase of survey distribution, researchers residing in 11 different administrative districts of Shanghai were invited to disseminate the online questionnaire, ensuring a broad geographic distribution of respondents. The survey distribution period was from June 1 to November 10, 2022, to ensure that participants had experienced the complete lockdown (from March 28 to June 1, 2022). The inclusion criteria were caregivers who: (1) had at least one child aged under 3 years, (2) lived with children aged under 3 years in Shanghai during the lockdown, and (3) signed an informed consent form. We excluded respondents who failed to provide complete information or complete the entire survey. All participants were informed of the purpose of this study and signed an informed consent form, which demonstrated their willingness to participate in the study. By November 10, 2022, a total of 552 survey responses had been collected, of which 477 met the inclusion criteria. Therefore, this study obtained a total of 477 valid questionnaires.

### Statistical analysis

Descriptive statistical analyses were employed to analyze the basic characteristics of children and their caregivers and families, and COVID-19-related information. Continuous variables were described using medians and interquartile ranges (IQR), while categorical variables were described using counts and percentages. The chi-square test was utilized to examine the differences in variable distributions between groups. Further, two logistic regression models were developed to investigate the factors influencing caregivers’ child healthcare preferences. The dependent variable in logistic regression models was caregivers’ child healthcare preference during the lockdown, treated as a categorical variable. Caregivers could choose one from four options: hospitals, nursery institutions, CHS, or others. In models 1 and 2, preference for hospitals and nursery institutions were used as the reference groups, respectively, while both models explored factors influencing caregivers’ child healthcare preference for CHS. Significant factors in the univariate tests were included in the logistic regression model. The model’s goodness-of-fit was evaluated using the Akaike Information Criterion (AIC), and multicollinearity was examined using the maximum variance inflation factor (VIF). All tests were two-tailed, and *P* < .05 was considered statistically significant. All analyses were conducted using SPSS software (Version 18.0; SPSS Inc, Chicago, USA) and R 4.3.3 (https://www.R-project.org/).

## Results

### Basic characteristics

In the sample, 93.1% of the caregivers were mothers, and 5.2% were fathers. The mean age was 32 years. Most had a bachelor’s degree, and the top two occupations were office worker and professional/technical personnel. Almost all were married; 40% had an annual family income of CNY 100,000–200,000; and most lived in Baoshan District.

Regarding each family’s youngest child (mean age: 18 months), nearly 30% had siblings. Of these, 5.5% were born prematurely, 4.4% had a low birth weight, and 16.6% had been admitted to neonatal intensive care. Nearly half of the participating children were fed formula milk (44.7%).

During the lockdown, four family members co-residing was the modal living situation (33.8%), 26.8% of the caregivers knew someone with COVID-19, over 60% worked from home, 57.9% felt some parenting anxiety, and 14.7% felt relatively or extremely high parenting anxiety. Nutrition and feeding were the most common issue related to early childhood development that caregivers experienced during lockdown, whereas difficulty in taking children out for healthcare due to fear of being infected by COVID-19 was the predominant difficulty related to healthcare-seeking. Most caregivers (81.3%) did not experience child healthcare unavailability. However, most said that their children had missed physical growth and developmental check-ups (75.1%) and vaccinations (66.2%).

During lockdown, 47.6% of caregivers preferred CHS, while 40.0% preferred hospital pediatrics (Table 1).

**Table 1:** Participants’ basic characteristics

Characteristic	*N* / Medium	% / Interquartile range
Caregiver’s role		
Father	25	5.2
Mother	444	93.1
Grandparents	6	1.3
Other guardian	2	0.4
Caregiver’s sex		
Male	29	6.1
Female	448	93.9
Caregiver’s age, years	32	30–35
20–29	113	23.7
30–39	333	69.8
40–49	29	6.1
≥ 50	2	0.4
Caregiver’s educational level		
Junior high school degree	14	2.9
Junior college degree	45	9.4
Bachelor’s degree	375	78.6
Master’s degree	37	7.8
Doctoral degree	6	1.3
Caregiver’s occupation		
Worker (e.g., factory worker/manual laborer)	14	2.9
Office worker	194	40.7
Business service personnel (e.g., waiter, salesman, driver)	6	1.3
Professional and technical personnel (e.g., healthcare provider, teacher)	122	25.6
Cadre of party and government organs and institutions, civil servant, village and neighborhood committee worker	13	2.7
Manager of state-owned enterprises (including middle and grass-roots managers)	8	1.7
Manager of private and foreign-funded enterprises (including mid-level and grass-roots managers)	13	2.7
Individual industrial and commercial enterprises	10	2.1
Freelancer	40	8.4
Agricultural laborer	1	0.2
Unemployed	45	9.4
Other	11	2.3
Caregiver’s marital status		
Single	1	0.2
Married	475	99.6
Divorced	1	0.2
Annual household income, CNY		
< 100,000	74	15.5
[100,000, 200,000)	191	40.0
[200,000, 300,000)	107	22.4
[300,000, 500,000)	79	16.6
[500,000, 800,000)	18	3.8
[800,000, 1,000,000)	4	0.8
≥ 1,000,000	4	0.8
Family residence, district		
Huangpu	42	8.8
Xuhui	5	1.0
Putuo	1	0.2
Hongkou	1	0.2
Yangpu	15	3.1
Minhang	19	4.0
Baoshan	374	78.4
Jiading	12	2.5
Pudong New Area	3	0.6
Jinshan	3	0.6
Songjiang	2	0.4
Whether the youngest child has siblings		
Yes	141	29.6
No	336	70.4
Age of the youngest child, months	18	10–29
≤ 1	11	2.3
(1, 3]	2	0.4
(3, 6]	25	5.2
(6, 8]	39	8.2
(8, 12]	95	19.9
(12, 18]	70	14.7
(18, 24]	74	15.5
(24, 30]	64	13.4
(30, 36]	97	20.3
Whether the youngest child was born prematurely (< 37 weeks)		
Yes	26	5.5
No	451	94.5
Whether the youngest child was born with low birth weight (< 2,500 kg)		
Yes	21	4.4
No	456	95.6
Whether the youngest child was admitted to the NICU		
Yes	79	16.6
No	398	83.4
Feeding pattern of the youngest child		
Breastfeeding	78	16.4
Mixed feeding	186	39.0
Formula feeding	213	44.7
Number of family members co-residing during lockdown		
2	23	4.8
3	105	22.0
4	161	33.8
5	139	29.1
6	39	8.2
7	6	1.3
8	4	0.8
Whether someone you know was infected with COVID-19 during lockdown		
Yes	128	26.8
No	349	73.2
Whether you worked from home during lockdown		
Yes	331	69.4
No	146	30.6
Whether you experienced the following early childhood development issues during lockdown		
Nutrition and feeding	198	41.5
Physical development (e.g., height, weight)	192	40.3
Other early childhood development (e.g., motor, language, socio-emotional)	166	34.8
Parent–child interaction (e.g., activities, education)	195	40.9
Other	23	4.8
Whether you experienced the following difficulties related to healthcare-seeking during lockdown		
Difficulty in accessing professional guidance from childcare providers in the CHS	188	39.4
Difficulty in accessing professional guidance from pediatricians in hospitals	174	36.5
Difficulty in taking children out for healthcare due to fear of being infected by COVID-19	231	48.4
Other	36	7.5
Level of parenting anxiety during lockdown		
None	107	22.4
A little bit	276	57.9
Uncertain	24	5.0
Relatively high	53	11.1
Extremely high	17	3.6
Whether you experienced unavailable healthcare demands during lockdown		
Yes	89	18.7
No	388	81.3
Whether your child missed physical growth and developmental check-ups during lockdown		
Yes	358	75.1
No	119	24.9
Whether your child missed vaccinations during lockdown		
Yes	316	66.2
No	161	33.8
Caregiver’s child healthcare preferences during lockdown		
CHS	227	47.6
Hospitals	191	40.0
Nursery institutions	52	10.9
Other	7	1.5

*Note* N, number; %, percent; NICU, neonatal intensive care unit; CHS, community health service system

### Univariate analysis of factors influencing caregivers’ child healthcare preferences

Significant differences were observed in the distribution of the following research factors among populations with different healthcare preferences: annual household income, youngest child’s age, issues with early childhood physical development, difficulty in accessing professional child healthcare guidance in CHS or hospitals, and missing physical growth and developmental check-ups (all *P*-values < 0.05; Table 2).

**Table 2:** Univariate analysis of factors influencing caregivers’ child healthcare preferences

Characteristic	Chi-square	*P*-value
Caregiver’s role	8.945	0.442
Caregiver’s sex	3.788	0.285
Caregiver’s age, years	11.705	0.230
Caregiver’s educational level	19.659	0.074
Caregiver’s occupation	42.368	0.127
Caregiver’s marital status	2.595	0.858
Annual household income, CNY	63.276	< 0.001^*^
Family residence, district	24.800	0.735
Whether the youngest child has siblings	2.342	0.505
Age of the youngest child, months	44.935	0.006^*^
Whether the youngest child was born prematurely (< 37 weeks)	0.938	0.816
Whether the youngest child was born with low birth weight (< 2,500 kg)	2.270	0.518
Whether the youngest child was admitted to the NICU	2.080	0.556
Feeding pattern of the youngest child	2.520	0.866
Number of family members co-residing during lockdown	26.678	0.085
Whether someone you know was infected with COVID-19 during lockdown	3.224	0.358
Whether you worked from home during lockdown	3.297	0.348
Whether you experienced the following early childhood development issues during lockdown		
Nutrition and feeding	6.296	0.098
Physical development (e.g., height, weight)	15.075	0.002^*^
Other early childhood development (e.g., motor, language, socio-emotional)	2.054	0.561
Parent–child interaction (e.g., activities, education)	4.133	0.247
Other	10.158	0.017^*^
Whether you experienced the following difficulties related to healthcare-seeking during lockdown		
Difficulty in accessing professional guidance from childcare providers in the CHS	11.665	0.009^*^
Difficulty in accessing professional guidance from pediatricians in hospitals	17.077	0.001^*^
Difficulty in taking children out for healthcare due to fear of being infected by COVID-19	4.817	0.186
Other	13.256	0.004^*^
Level of parenting anxiety during lockdown	4.071	0.982
Whether you experienced unavailable healthcare demands during lockdown	2.111	0.550
Whether your child missed physical growth and developmental check-ups during lockdown	7.827	0.050^*^
Whether your child missed vaccinations during lockdown	6.777	0.079

Note^*^Indicates statistically significant results (*p* < .05); NICU, neonatal intensive care unit; CHS, community health service system

### Multivariate analysis of factors influencing caregivers’ child healthcare preferences

In logistic regression model 1, caregivers’ child healthcare preference for hospitals was taken as the reference group. Therefore, this model analyzed factors influencing caregivers’ child healthcare preference for CHS over hospitals (Table 3). The AIC of this model was 551.68 and the VIF value was 1.31, indicating the absence of multicollinearity. Compared to families with an annual income of < 100,000 CNY, caregivers with a family annual income of 200,000–300,000 CNY were more inclined to choose CHS (*OR* = 2.426). Caregivers who experienced healthcare-seeking-related difficulties in accessing professional hospital guidance showed a positive correlation with a preference for hospitals (*OR* = 0.293). Conversely, caregivers who experienced healthcare-seeking-related difficulties in accessing professional guidance from CHS professionals were more inclined towards CHS rather than hospitals (*OR* = 2.419).

**Table 3:** Logistic model 1: preference for CHS vs. Preference for hospitals

Characteristic	OR	95% Wald confidence limit		*P*-value
		Lower	Upper	
Annual household income, CNY				
< 100,000	ref	ref	ref	ref
[100,000, 200,000)	0.962	0.514	1.800	0.903
[200,000, 300,000)	2.426	1.188	5.018	0.016^*^
[300,000, 500,000)	1.345	0.647	2.809	0.428
[500,000, 800,000)	0.673	0.182	2.403	0.540
[800,000, 1,000,000)	> 999.999	< 0.001	> 999.999	0.988
≥ 1,000,000	< 0.001	< 0.001	> 999.999	0.992
Youngest child’s age, months				
≤ 1	ref	ref	ref	ref
(1, 3]	< 0.001	< 0.001	> 999.999	0.992
(3, 6]	1.103	0.185	7.328	0.915
(6, 8]	1.552	0.274	9.527	0.635
(8, 12]	1.81	0.362	10.371	0.476
(12, 18]	1.341	0.263	7.770	0.726
(18, 24]	1.865	0.368	10.823	0.458
(24, 30]	0.689	0.132	4.096	0.663
(30, 36]	1.582	0.319	9.005	0.580
Whether you experienced early childhood physical development issues during lockdown				
No	ref	ref	ref	ref
Yes	1.232	0.747	2.041	0.414
Whether you experienced healthcare-seeking difficulties in accessing professional guidance from childcare providers in CHS^a^ during lockdown				
No	ref	ref	ref	ref
Yes	2.419	1.406	4.268	0.002^*^
Whether you experienced healthcare-seeking difficulties in accessing professional guidance from hospitals				
No	ref	ref	ref	ref
Yes	0.293	0.166	0.502	< 0.001^*^
Whether your child missed physical growth and developmental check-ups				
No	ref	ref	ref	ref
Yes	1.472	0.873	2.490	0.148

Note^*^Indicates statistically significant results (*p* < .05); ^a^CHS, community health service system; OR, odds ratio

In logistic regression model 2, caregivers’ child healthcare preference for nursery institutions was taken as the reference group. Therefore, this model analyzed factors influencing caregivers’ child healthcare preference for CHS over nursery institutions (Table 4). The AIC of this model was 249.87 and the VIF value was 1.33, indicating the absence of multicollinearity. Compared to children aged ≤ 1 year, caregivers with children aged between 8 and 12 months were more inclined to choose CHS over nursery institutions (*OR* = 23.820). Moreover, caregivers experiencing early childhood physical development issues had stronger preference for CHS than for nursery institutions (*OR* = 1.989).

**Table 4:** Logistic model 2: preference for CHS vs. preference for nursery institutions

Characteristic	OR	95% Wald confidence limit		*P*-value
		Lower	Upper	
Annual income, CNY				
< 100,000	ref	ref	ref	ref
[100,000, 200,000)	2.391	0.833	6.923	0.104
[200,000, 300,000)	3.561	0.116	11.329	0.079
[300,000, 500,000)	4.463	0.124	17.616	0.058
[500,000, 800,000)	0.952	0.195	4.751	0.951
[800,000, 1,000,000)	> 999.999	< 0.001	> 999.999	0.988
≥ 1,000,000	< 0.001	< 0.001	> 999.999	0.992
Youngest child’s age, months				
(1, 3]	ref	ref	ref	ref
(3, 6]	5.904	0.395	165.835	0.214
(6, 8]	13.221	0.911	365.278	0.068
(8, 12]	23.820	2.078	299.950	0.009^*^
(12, 18]	7.225	0.736	64.427	0.071
(18, 24]	3.069	0.344	23.297	0.274
(24, 30]	1.115	0.121	8.661	0.917
(30, 36]	1.809	0.214	12.699	0.549
Whether you experienced early childhood physical development issues during lockdown				
No	ref	ref	ref	ref
Yes	1.989	1.425	4.956	0.025^*^
Whether you experienced healthcare-seeking difficulties in accessing professional guidance from childcare providers in CHS^a^ during lockdown				
No	ref	ref	ref	ref
Yes	1.647	0.630	4.487	0.317
Whether you experienced healthcare-seeking difficulties in accessing professional guidance from hospitals				
No	ref	ref	ref	ref
Yes	0.505	0.178	1.406	0.192
Whether your child missed physical growth and developmental check-ups				
No	ref	ref	ref	ref
Yes	1.003	0.462	2.112	0.994

Note^*^Indicates statistically significant results (*p* < .05); ^a^CHS, community health service system; OR, odds ratio

## Discussion

This study explored caregivers’ child healthcare preferences and the factors that influenced them for children aged under 3 years during the large-scale, long-term COVID-19 lockdown in Shanghai. It found new evidence of caregivers’ child healthcare preferences and related factors during pandemic lockdown based on the SEM. The results revealed that caregivers preferred CHS (47.6%) followed by hospital pediatricians (40.0%) during the COVID-19 lockdown in Shanghai. Specifically, caregivers were more likely to prefer CHS if they had an annual household income of CNY 200,000–300,000, experienced healthcare-seeking-related difficulties in accessing professional guidance from CHS professionals, experienced early childhood physical development issues, and their youngest child was aged 8–12 months. Conversely, caregivers were more likely to prefer hospitals if they had experienced healthcare-seeking-related difficulties in accessing professional guidance from hospital pediatricians.

This study has several limitations. First, despite efforts to increase the participant range across the 11 Shanghai districts, selection bias may have occurred due to an unbalanced distribution of the questionnaires. Second, Shanghai is considerably different from other Chinese regions developmentally, economically, and medically, limiting the generalizability of this study’s findings. Third, although we requested caregivers to provide their child healthcare preferences during lockdown with highlighted hints in the questionnaire, the provided preferences might still be influenced by post-lockdown attitudes because some surveys were completed nearly six months after the end of lockdown. This may have introduced recall bias. Fourth, when designing the questionnaire to investigate children’s feeding patterns, age-appropriate solid food choices were not considered. These should be included in similar studies in the future.

This study found that, during lockdown, compared to hospitals, caregivers were more likely to choose CHS for their young children. The proportions for CHS, hospitals, and nursery institutions were 47.6%, 40.0%, and 10.9%, respectively. Generally, this finding contrasts with pre-pandemic national and regional surveys in China. The Chinese healthcare delivery system has a prominent “upside-down” problem in resource allocation. That means that under normal circumstances (pre-pandemic), caregivers showed a preference for tertiary hospitals due to their better healthcare resources and professional capabilities [32], as indicated by a national survey conducted in 2015–2016 [33]. The child health-seeking data in Shanghai also supported the existence of this problem. For example, in 2019, tertiary hospitals, secondary hospitals, and CHS in Shanghai accounted for 62.8%, 26.2%, and 11.0% of the total pediatric outpatient and emergency visits, respectively [34]. This trend persisted in some areas, such as Guangzhou, even during lockdown [35]. However, during the lockdown in Shanghai, this “upside-down” problem was reversed and the preference for CHS became much stronger. This could be attributed to the well-established primary healthcare system for young children and the close connections between caregivers and this system [36, 37]. For example, mandatory health records are created for every newborn, including regular physical check-ups and follow-up services [38]. Furthermore, Shanghai’s primary healthcare system development ranks among the top healthcare systems nationwide [39], with most families registered with community doctors [40]. These close connections allow caregivers to directly contact family doctors via mobile phones or messaging apps to learn about available pediatric services in their community in a timely manner, which was particularly helpful during the pandemic lockdown [41]. Therefore, during lockdowns, policymakers should recognize this positive tendency and allocate more resources to enhancing the quality of CHS child healthcare services [42, 43].

Additionally, this study found that caregivers who were concerned about their children’s early physical development were more inclined to choose CHS. In China, physical growth and developmental issues among young children are a serious concern. By 2020, the prevalence rates for growth stunting, malnutrition, and overweight among children under 5 years old were 4.7%, 1.9%, and 8.3%, respectively [5]. In Shanghai, the CHS plays a crucial role in providing regular physical check-ups for children aged 0–3 years. Consequently, the continuity and regularity of these check-ups have strengthened the bond between CHS and caregivers, explaining why caregivers dealing with their children’s developmental concerns under pandemic lockdown measures leaned towards CHS. Furthermore, caregivers with a family annual income of 200,000-300,000 CNY and those with children aged 8–12 months were more likely to choose CHS. Caregivers in this income group might have a greater awareness of utilizing primary child healthcare and may be reluctant to incur additional costs from private childcare institutions [44]. In comparison, caregivers with lower annual household incomes (e.g., less than 100,000 CNY or 100,000-200,000 CNY) often lack awareness of child health services [45]. Many of these caregivers are migrants who experience lower levels of social adaptation [46], leading them to place excessive trust in higher-level hospitals. Consequently, they prefer visiting hospital pediatric departments for their children’s illness, rather than utilizing CHS for routine child healthcare [47]. Moreover, parents with children aged 8–12 months might seek healthcare assistance from CHS more frequently due to mandatory check-up and follow-up appointments, particularly during lockdown.

This study found a positive correlation between caregivers’ preference for hospitals and healthcare-seeking-related difficulties in accessing pediatric hospital services, as well as between a preference for CHS and healthcare-seeking-related difficulties in accessing services from CHS childcare providers. These findings might be related with the inability of cross-sectional studies to determine the causal relationship between two factors. It indicated that caregivers’ preference for a particular healthcare institution led to actual difficulties in obtaining healthcare during lockdown rather than that such healthcare-seeking-related difficulties led to this preference. In 2022, outpatient and emergency pediatric visits in CHS accounted for only 9.1% of all such visits [34], significantly lower than the proportion of caregivers’ preference for CHS in this study. This suggests that a substantial proportion of the demand for CHS was unmet. Yet, since caregivers were required to provide their child healthcare preferences during lockdown, it is plausible that some persisted with their preferred healthcare institutions despite facing difficulties. A United States study showed that despite lockdown restrictions and increased availability of digital interventions, parents still favored comprehensive in-person behavioral interventions by physicians [48], which suggested that child healthcare preferences are hard to shift. Therefore, redirecting caregivers’ child healthcare needs to other institutions during lockdown might not be appropriate, as caregivers might persist in their preferences, and this might cause them to miss necessary healthcare services. As a result, it might be essential to optimize child healthcare resources in different healthcare institutions based on caregivers’ perceptions of difficulties in accessibility. For example, based on experiences in China, many CHS and hospital pediatricians may change roles to engage in epidemic prevention during pandemics. This shift complicates children’s access to medical resources [49, 50]. The government and healthcare authorities should develop a reserve team for public health emergencies in the post-pandemic era to ensure routine healthcare activities, especially for children. This team could include medical students, social volunteer groups, and government staff [51, 52].

## Conclusion

This study highlights the distribution of caregivers’ healthcare preferences related to children aged 0–3 years in Shanghai during the COVID-19 lockdown, as well as the factors influencing them. During such pandemic lockdowns, it is imperative for the government to ensure the accessibility of pediatric healthcare services across all healthcare institutions and to appropriately increase the resource allocation to CHS according to caregivers’ child healthcare preferences. Special attention should be given to the healthcare needs for CHS among families with an annual income of 200,000-300,000 CNY, with children aged 8–12 months, or experiencing early childhood physical development issues. This emphasizes the need to optimize resource allocation and focus on the healthcare needs of families with specific demographics, which is crucial for maintaining and enhancing the accessibility and quality of child healthcare services during challenging public health emergencies.

### Electronic supplementary material

Below is the link to the electronic supplementary material.

Supplementary Material 1

## Data Availability

The datasets used during the current study are available from the corresponding author on reasonable request.
