# Supplementary material for: Characteristics and influencing factors of caregivers’ healthcare preferences for young children under COVID-19 lockdown: a cross-sectional study in Shanghai, China
Source: BMC Prim Care. 2024 Jul 20;25:263. doi: 10.1186/s12875-024-02484-4 (PMC11264815; doi:10.1186/s12875-024-02484-4)
Supplement: Supplementary file 1 — Supplementary Material 1 [file 12875_2024_2484_MOESM1_ESM.pdf]

A Survey on the Caregivers' Child Healthcare Preferences for Children under 3 years during COVID-19 Lockdown

Dear Sir/Madam,

Thank you very much for your participation!

In March, 2022, the COVID-19 outbreak in Shanghai led to the full lockdown, which has brought great changes to our lives and work. Because of the pandemic, families with young children have especially been facing challenges of healthcare behaviors and utilization. Caregivers' preferences may also change as never before because of lockdown measures.

In the face of these impacts, this study aims to clarify the healthcare preferences among families with young children. It hopes to rationally optimize resource allocation adjustment of related support policies, and guarantee of healthcare services for early childhood development in future situations similar to pandemic lockdown..

The questionnaire was completed anonymously for academic research purposes only. All data can only be seen by the researchers to ensure the security of your information. Please answer truthfully, there is no right or wrong answer. I wish you good health and family peace!

1. Caregiver's role

☐Father ☐Mother ☐Grandparents ☐Other guardian

2. Caregiver's sex

☐Male ☐Female

3. Caregiver's birth year: ______________

4. Caregiver's educational level

☐Junior high school degree ☐Junior college degree
☐Bachelor's degree ☐Master's degree
☐Doctoral degree

5. Caregiver's occupation

☐Worker (e.g., factory worker/manual laborer)
☐Office worker
☐Business service personnel (e.g., waiter, salesman, driver)
☐Professional and technical personnel (e.g., healthcare provider, teacher)
☐Cadre of party and government organs and institutions, civil servant, village and neighborhood committee worker
☐Manager of state-owned enterprises (including middle and grass-roots managers)
☐Manager of private and foreign-funded enterprises (including mid-level and grass-roots managers)
☐Individual industrial and commercial enterprises
☐Freelancer

☐Agricultural laborer
☐Unemployed
☐Other

6. Caregiver's marital status
☐Single ☐Married ☐Divorced

7. Annual household income, CNY
☐ <100,000 ☐ [100,000, 200,000)
☐ [200,000, 300,000) ☐ [300,000, 500,000)
☐ [500,000, 800,000) ☐ [800,000, 1,000,000)
☐ ≥1,000,000

8. Family residence, district: __________

9. Whether the youngest child has siblings?
☐Yes ☐No

10. Age of the youngest child, months: _________

11. Whether the youngest child was born prematurely (<37 weeks)?
☐Yes ☐No

12. Whether the youngest child was born with low birth weight (<2,500 kg)?
☐Yes ☐No

13. Whether the youngest child was admitted to the NICU?
☐Yes ☐No

14. What feeding pattern has your youngest child experienced or is experiencing?
☐Breastfeeding ☐Mixed feeding ☐Formula feeding

15. Number of family members co-residing during lockdown: _______

16. Whether someone you know was infected with COVID-19 during lockdown?
☐Yes ☐No

17. Whether you worked from home during lockdown?
☐Yes ☐No

18. Whether you experienced the following early childhood development issues during lockdown? (Multiple choices)
☐ Nutrition and feeding (e.g., nutrition, breastfeeding, food types)
☐Child physical development (e.g., height, weight)

☐ Other early childhood development (e.g., gross motor, language, socioemotional)
☐Parent–child interaction (e.g., how to accompany child, how to educate child)
☐Other

19. Whether you experienced the following difficulties related to healthcare-seeking during lockdown? (Multiple choices)
☐Difficulty in accessing professional guidance from childcare providers in the CHS
☐Difficulty in accessing professional guidance from pediatricians in hospitals
☐Difficulty in taking children out for healthcare due to fear of being infected by COVID-19
☐Other

20. Please evaluate your level of parenting anxiety due to this pandemic lockdown.
☐None ☐A little bit
☐Uncertain ☐Relatively high
☐Extremely high

21. Whether you experienced unavailable healthcare demands due to this pandemic lockdown?
☐Yes ☐No

22. Whether your child missed physical growth and developmental check-ups due to this pandemic lockdown?
☐Yes ☐No

23. Whether your child missed vaccinations due to this pandemic lockdown?
☐Yes ☐No

24. After experiencing this pandemic lockdown, what is your first choice when your child needs healthcare services?
☐CHS ☐Hospitals
☐Nursery institutions ☐Other
